# Layperson-Facilitated Internet-Delivered Cognitive Behavioral Therapy for Homebound Older Adults With Depression: Protocol for a Randomized Controlled Trial

**DOI:** 10.2196/44210

**Published:** 2023-02-22

**Authors:** Jay Kayser, Xu Wang, Zhenke Wu, Asha Dimoji, Xiaoling Xiang

**Affiliations:** 1 School of Social Work University of Michigan Ann Arbor, MI United States; 2 Computer Science and Engineering University of Michigan Ann Arbor, MI United States; 3 Department of Biostatistics University of Michigan Ann Arbor, MI United States; 4 College of Literature, Science, and Arts University of Michigan Ann Arbor, MI United States

**Keywords:** depression, depressive, gerontology, gerontological, geriatric, psychotherapy, older adult, elder, home based, community based, community living, homebound, low-income, eHealth, mHealth, digital mental health, internet intervention, iCBT, CBT, mental health, cognitive behavioral therapy, internet based, internet delivered

## Abstract

**Background:**

Depression in older adults has serious biological, psychological, and social consequences. Homebound older adults experience a high burden of depression and significant barriers to accessing mental health treatments. Few interventions to address their specific needs have been developed. Existing treatment modalities can be challenging to scale up, are not tailored to unique population concerns, and require significant staffing support. Technology-assisted, layperson-facilitated psychotherapy has the potential to overcome these challenges.

**Objective:**

The aim of this study is to assess the efficacy of a layperson-facilitated internet-delivered cognitive behavioral therapy program tailored for homebound older adults. The novel intervention, Empower@Home, was developed based on user-centered design principles and partnerships between researchers, social service agencies, care recipients, and other stakeholders serving low-income homebound older adults.

**Methods:**

This 2-arm, 20-week pilot randomized controlled trial (RCT) with a waitlist control crossover design aims to enroll 70 community-dwelling older adults with elevated depressive symptoms. The treatment group will receive the 10-week intervention immediately, whereas the waitlist control group will cross over and receive the intervention after 10 weeks. This pilot is part of a multiphase project involving a single-group feasibility study (completed in December 2022). This project consists of a pilot RCT (described in this protocol) and an implementation feasibility study running in parallel with the pilot RCT. The primary clinical outcome of the pilot is the change in depressive symptoms after the intervention and at the 20-week postrandomization follow-up. Additional outcomes include acceptability, adherence, and changes in anxiety, social isolation, and quality of life.

**Results:**

Institutional review board approval was obtained for the proposed trial in April 2022. Recruitment for the pilot RCT began in January 2023 and is anticipated to end in September 2023. On completion of the pilot trial, we will examine the preliminary efficacy of the intervention on depression symptoms and other secondary clinical outcomes in an intention-to-treat analysis.

**Conclusions:**

Although web-based cognitive behavioral therapy programs are available, most programs have low adherence and very few are tailored for older adults. Our intervention addresses this gap. Older adults, particularly those with mobility difficulties and multiple chronic health conditions, could benefit from internet-based psychotherapy. This approach can serve a pressing need in society while being cost-effective, scalable, and convenient. This pilot RCT builds on a completed single-group feasibility study by determining the preliminary effects of the intervention compared with a control condition. The findings will provide a foundation for a future fully-powered randomized controlled efficacy trial. If our intervention is found to be effective, implications extend to other digital mental health interventions and populations with physical disabilities and access restrictions who face persistent disparities in mental health.

**Trial Registration:**

ClinicalTrials.gov NCT05593276; https://clinicaltrials.gov/ct2/show/NCT05593276

**International Registered Report Identifier (IRRID):**

PRR1-10.2196/44210

## Introduction

### Background

Depression in older adults has serious consequences, including worsened physical health and chronic disease progression [1-3], increased functional limitations and disabilities [4,5], institutionalization [6], and premature mortality due to comorbid conditions and suicide [7,8]. Low-income and homebound older adults are especially at high risk for experiencing depression. In one US representative sample, estimates for rates of depression in homebound older adults were approximately 40% [9]. Further complicating this burden of depression is a recent steep increase in the number of homebound older adults, likely in part driven by COVID-19 [10].

Although evidence-based pharmacological and nonpharmacological treatments exist, not all options are readily accessible to all populations of older adults [11]. Low-income and homebound older adults face significant access barriers to mental health treatments due to mobility, transportation, and financial difficulties. In addition, few providers have specialized training in working with older populations [12,13]. Given the lack of service providers, high rates of depression in specific subpopulations of older adults, and demographic trends toward a higher number of homebound older adults, interventions that are both accessible and scalable are urgently needed.

Digital mental health interventions have the potential to address the gaps in mental health treatment faced by low-income and homebound older adults. Affirming this, a recent national panel of expert stakeholders recommended that digital mental health interventions be broadly adopted and that insurers expand their coverage and reimbursement [14]. Cognitive behavioral therapy (CBT) is an evidence-based form of psychological treatment and has been adapted to multiple age groups [15-17] as well as medical and social contexts [18,19]. CBT is well suited for digital-based delivery, given its highly structured format and skill-based learning [20]. Hundreds of clinical trials have taken place in the last decade to test internet-delivered CBT (iCBT) and presented robust findings supporting the acceptability and effectiveness of this treatment modality [21-25]. iCBT is convenient and self-paced, less expensive than traditional psychotherapy, and scalable due to content delivery automation.

Research has consistently shown that tailoring treatment to specific populations, contexts, and settings is associated with increased uptake, acceptability, and sustainability [26,27]. In the case of older adults, procedural and content modifications to CBT have been discussed that incorporate differences in thinking styles in older adults and age-related adjustments [28]. However, when packaged for internet delivery, empirically supported iCBT programs often employ a one-size-fits-all approach, and very few have been designed to consider the needs of older adults [29-31]. In general, older adults are underrepresented in iCBT trials. A review found that only 3% of iCBT trial participants were older adults [32]. Since then, older adults’ participation has increased. However, a recent systematic review found that older adults are still largely under-represented as participants in rigorously designed studies [25].

Recent studies involving homebound older adults have shown that modifications to both contents (eg, adding aging-related themes) and web interface (website or app) are needed to improve adherence and engagement with iCBT [33,34]. These studies suggest that older adults had difficulty applying lessons when presented with content that failed to address aging-related topics and were less tolerant to poor usability issues, possibly due to a combination of age-related changes (eg, cognitive, vision, motor control, speech, and hearing), physical disabilities, and their lack of familiarity with modern technology. Most well-established iCBT programs contain a large amount of text, are highly didactic, and are hosted on interfaces that are not age-friendly (eg, small text and buttons). Usability issues and technical challenges are frequently reported, which can lower adherence and ultimately reduce treatment effects. The lack of tailored solutions may be more problematic for homebound older adults, who tend to be older, less tech-savvy, have more cognitive and functional limitations, and are more likely to experience usability issues [35].

### Objective

The primary aim of this study is to evaluate the efficacy of a 9-session iCBT program in treating depression in low-income homebound older adults. Additional outcomes and potential covariates will be captured, including anxiety, loneliness, and health-related quality of life.

A lack of iCBT products suitable for homebound older adults reflects and contributes to the digital divide experienced by them. Barring treatment innovation, older adults will continue to be left behind and face persistent mental health disparities related to access. This study aims to fill this void by testing the preliminary effects of a novel iCBT program designed for homebound older adults.

### Intervention Model

Empower@Home is an iCBT program designed to treat the core symptoms of depression in older adults. It consists of 9 web-based sessions grounded in CBT principles and homework assignments (ie, weekly practices) made available over 10 weeks. Each lesson is presented as a series of short videos with voice-overs that include didactic text, images of diverse older adults, inspirational quotes, and interactive exercises. Sessions are written at an eighth-grade reading level, and all spoken content is captioned. The weekly home practice tasks are modest and typically require 20 minutes or less of commitment from the user. A workbook with summarized session content and home practices is also provided to participants.

The sessions are enhanced with an animated case story series featuring a 74-year-old homebound woman named Jackie. The animated story is embedded within each session, like an episode of a television show. This content serves to reinforce core skills and techniques taught. Prior research has found that including short multimedia entertainment is a useful approach for communicating learning objectives in psychoeducational materials [36].

The session scripts of Empower@Home were developed by a team of researchers, clinicians, older adults, and social service providers via a co-design process. The web platform that delivers Empower@Home is a custom learning management system built using state-of-the-art, agile development processes. All interface features are compatible with accessibility standards and current best practices for making an age-friendly user interface and experience design [37]. For example, the main user interface features large buttons, text, icons with text descriptions, high-contrast color schemes, and intuitive navigation.

Informed by the Efficiency Model of Support [38], support from trained laypersons called Empower Coaches supplements the core web-based sessions of Empower@Home. Human support aims at increasing a participant’s ability to use the technology to obtain the intended treatment outcome [38]. Previous studies have shown that supported iCBT is more effective and associated with better adherence than unsupported interventions [39-41]. However, support mechanisms are generally underspecified and understudied, and it is not entirely clear what type and intensity of support are needed for homebound older adults to engage in iCBT successfully.

Informed by our previous research and the Efficiency Model of Support, we provide weekly check-ins with a supportive guide (ie, Empower Coach). The weekly check-ins are Coach-initiated contacts designed to prevent and address potential failure points, including engagement, working knowledge of the iCBT platform, and implementation of iCBT. All weekly check-ins are telephone-based. The Empower Coaches are laypersons without specialized mental health training or independent clinical licensure. The support content includes simple feedback, encouragement, homework review, and assistance with self-help tools without additional traditional therapy. Coaching sessions are highly individualized to meet the particular needs of the participants. On the basis of participants’ preferences, they may use the weekly check-in sessions to go over the web-based program with their coaches, whereas others complete the web-based portion on their own before the phone-based check-in sessions. Coaching sessions can range from 5 minutes to an hour per week, depending on the participants’ needs and preferences.

Coach training involves reviewing all web-based sessions of Empower@Home (takes approximately 4-5 hours to complete), a self-directed asynchronous web-based training (approximately 1.5 hours), and a synchronous training workshop (approximately 3 hours). The total training burden is approximately 9-10 hours per coach.

## Methods

### Preliminary Study

The procedures described in this protocol, including recruitment, assessments, coaching calls, and device shipments and returns, have been tested in a completed single-group uncontrolled feasibility study. Findings from the single-group study have not yet been published, but preliminary analysis shows that the intervention is feasible and acceptable, with high satisfaction ratings, and is associated with significant within-group reductions in depressive symptoms and several secondary clinical outcomes. Study procedures for the pilot randomized controlled trial (RCT) are refined based on lessons learned from the feasibility pilot.

### Study Setting and Aims

The study will be managed from the University of Michigan in collaboration with community agencies serving older adults. Participants will reside throughout Michigan in both rural and urban areas. All participants will be 60 years or older and are expected to be predominantly low income. Participants will complete the intervention in their own homes, with all assessments and technological support provided remotely by phone.

### Eligibility Criteria

To be eligible for the trial, participants must be at 60 years or older and have at least mild depression (score of ≥8 on the Patient Health Questionnaire-9 [PHQ-9]). Participants will be excluded if they (1) have probable cognitive impairment based on the Blessed Orientation-Memory-Concentration Test (score ≥10), (2) do not speak English, (3) have moderate to high risk of suicide based on the Columbia-Suicide Severity Rating Scale, (4) have a terminal illness or are in unstable physical health with a high risk of repeated hospitalizations within the next 3 months, (5) have a visual impairment based on self-reported inability to use a screen with correction, (6) have a current or recent history of substance use based on a score of ≥2 on the CAGE-AID Substance Abuse Screening Tool, (7) have a psychotic disorder based on self-report, or (8) are currently receiving psychotherapy. The study will not exclude participants based on racial or gender identification. Completing the intervention will require a tablet and internet accessibility. Participants are not required to have a computer or internet access; those who lack 1 or both resources will be provided a tablet and access to the internet at no cost during the active intervention period.

### Recruitment and Compensation

Participants will be recruited into the study on a rolling basis and will be drawn from multiple community agencies as well as other public places, including social media and local news outlets. The social service agencies that participated in this study’s intervention development and implementation feasibility phase were predominantly Area Agencies on Aging based throughout Michigan. These agencies serve older adults by offering services to enable their clients to maintain independence. These services may involve case management, caregiver services, and meals on wheels. These agencies will refer participants to the study based on their judgment of the appropriateness of the intervention for their clients.

After receiving referrals, study personnel will contact potential participants via phone to screen for eligibility. The initial screening is expected to take 20 minutes to complete. Participants will provide recorded verbal consent. Those who decline to participate will be referred back to their case managers or agency when possible. Advertisements placed via social media (eg, Facebook and Nextdoor) and through research registries will also allow participants to self-refer and be screened for eligibility.

Those who qualify for the trial will be paid up to US $100. Compensation is determined based on the number of assessments completed. Partial compensation is available (eg, participants who complete only the baseline assessment will receive US $30).

### Study Design

Eligible and consented participants will be assigned either to an active treatment or a waitlist condition group that will cross over to the intervention at 10 weeks. A computer-generated random sequence will be used for assignment into either condition at a ratio of 1:1. Given the content of the intervention and study design, blinding the participant and evaluator to the condition will not be feasible.

**Figure 1:**
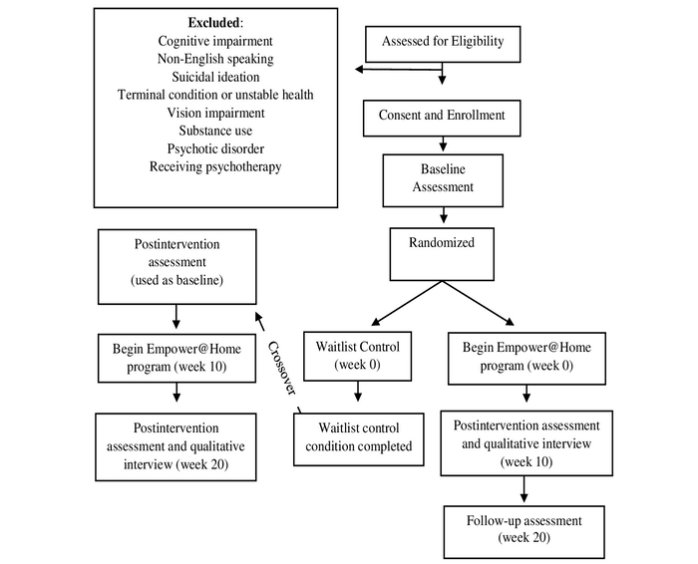
Study design.

All participants will be assessed using the same schedule. All will be invited to complete the comprehensive baseline assessment, postintervention or control assessment at week 10, and a follow-up assessment at week 20. Participants on the waitlist control will receive the intervention after their post-test at week 10 (Figure 1).

### Waitlist Condition

The attention control waitlist condition involves a comparable amount of phone contact to the intervention condition, with roughly 20 minutes of companionship calls per week. However, in the attention control, participants will not be exposed to CBT-related content. The purpose of companionship calls is to provide socialization and contact with supportive individuals without introducing structured therapeutic content.

### Discontinuation

Consented participants may still be deemed ineligible during study participation if they develop significant clinical worsening of depression, suicidal ideation, or other circumstances (eg, terminal illness or prolonged hospitalization) that preclude their ongoing involvement with the trial. Participants will be consulted regarding potential removal. Should a participant report suicidal ideation, a protocol will be in place that involves the participation of the study principal investigator and a clinical social worker. Should a participant’s circumstance interfere with their ongoing participation in the trial, the study team will discuss the risks and benefits of the participant’s ongoing involvement with the trial until a consensus is reached. If there is a clinically significant worsening of depressive symptoms, considerable effort will be made to expedite entry into the best available treatment outside the research protocol.

### Clinical Management and Storage

All data related to the study (screening, pretest, coaching sessions, posttest, and follow-up) will be entered directly by project staff into REDCap [42]. During the active intervention, participants will be asked to fill out the PHQ-9 every other week, administered as a part of the web-based therapy lesson. These assessment data are stored in a HIPAA (Health Insurance Portability and Accountability Act)–compliant database hosted by the University of Michigan.

### Measures

All measures are presented in Table 1. The primary clinical outcome in the study will be depressive symptoms as measured by the PHQ-9. The PHQ-9 is widely used in clinical research and has been used to evaluate older adults with medical and psychiatric illnesses in and outside of health care settings [43,44]. The PHQ-9 has been found to perform comparably to the Geriatric Depression Scale in identifying depression in older adults [45]. Scores on the PHQ-9 can range from 0 to 27 and indicate minimal, mild, moderate, moderately severe, or severe depression symptom severity. Sample items include “In the last week, have you had little interest or pleasure in doing things?” and “In the last two weeks, have you been feeling down, depressed, or hopeless?” Respondents indicate answers on a 4-point Likert scale ranging from “not at all” to “nearly every day.” In primary care patients, the measure has a sensitivity of 88% and a specificity of 88% [43]. The PHQ-9 will be administered at initial screening, baseline, after intervention, and 20-week follow-up. In addition, participants will complete a PHQ-9 self-assessment built into the web-based sessions every other week. To mimic the additional assessment patterns in the treatment group, participants in the control condition will also be administered the PHQ-9 every other week via phone during the 10-week waiting period.

**Table 1:** Study measures and time points.

Variable	Assessment or activity	Screening	Baseline	Postrandomization			Follow-up
				Biweekly	Post		
Depression	PHQ-9^a^	✓	✓	✓	✓	✓	
Anxiety	GAD-7^b^		✓		✓	✓	
Social isolation	PROMIS-SI 8a^c^		✓		✓	✓	
Health self-assessment	PROMIS-GH^d^		✓		✓	✓	
Well-being and quality of life	EQ-5D-5L		✓		✓	✓	
Skill acquisition	CBTSQ^e^		✓		✓	✓	
Acceptability	Modified TEI^f^				✓		
Adherence	Session completion				✓		
Participant background	Sociodemographic, health status, and physical functioning	✓	✓				

^a^PHQ-9: Patient Health Questionnaire-9.

^b^GAD-7: Generalized Anxiety Disorder-7.

^c^PROMIS-SI 8a: Patient-Reported Outcomes Measurement Information System–Social Isolation 8a.

^d^PROMIS-GH: Patient-Reported Outcomes Measurement Information System–General Health.

^e^CBTSQ: Cognitive-Behavioral Therapy Skills Questionnaire.

^f^TEI: Treatment Evaluation Inventory.

Anxiety will be assessed with the General Anxiety Disorder-7, which has been validated in populations of older adults [46]. Sample items include “How often have you been bothered by feeling nervous, anxious, or on edge in the last two weeks?” and “How often have you been bothered by worrying too much about different things in the last two weeks?” Like the PHQ-9, respondents answer on a 4-point Likert scale ranging from “not at all” to “nearly every day.” In primary care patients, the measure has a sensitivity of 89% and a specificity of 82% [47].

Social isolation will be measured using the Patient-Reported Outcomes and Measures Information System–Social Isolation (PROMIS-SI 8a). This measure has been used extensively in older adults with chronic health conditions and depression [48-50]. This 8-item measure uses a 5-point Likert scale with responses ranging from “always” to “never.” Sample items include “I feel isolated from others” and “I feel that people avoid talking to me.” The PROMIS-SI has high internal consistency (Cronbach α=.91) [51].

The PROMIS–General Health (PROMIS-GH) will be used as a global measure of health and health-related quality of life. This 10-item measure uses a 5-point Likert scale ranging from “excellent” to “poor.” It contains items such as “In general, would you say your health is...?” and “In general, how would you rate your satisfaction with your social activities and relationships…?” The PROMIS-GH has been validated in older adults with chronic health conditions, demonstrating moderate internal reliability (ordinal α=.82-.88) [52].

Quality of life will be measured using the EQ-5D-5L. Respondents answer on a 5-point Likert scale ranging from “I have no problems with…” to “I am unable to…/I have extreme…” They rate their experience in 5 life areas (mobility, self-care, usual activities, pain, and anxiety or depression). It also includes an overall health ranking on a scale of 0-100. In a systematic review including 99 studies that included a diverse range of medical conditions, the EQ-5D-5L exhibited overall excellent psychometric properties [53].

To explore the role of CBT skill acquisition, participants will complete the Cognitive-Behavioral Therapy Skills Questionnaire. This validated 16-item questionnaire assesses a respondent’s ability to apply cognitive restructuring, behavioral activation, and other CBT-related skills [54]. Participants are asked how often they currently do sample activities, such as making plans over the weekend, motivating themselves to do things, and socializing even though they do not want to. Items are answered on a 5-point Likert scale ranging from “I don’t do this” to “I always do this.”

Acceptability will be assessed by the number of sessions participants completed (ie, adherence) and the modified Treatment Evaluation Inventory. This 11-item inventory has been previously used to evaluate the acceptability of cognitive therapy for depression interventions among older adults and has good convergent validity and internal consistency (α=.92) [55]. The measure will be administered after the intervention. The measure is answered on a 5-point Likert scale ranging from “never” to “always.”

### Qualitative Interviews

After completing the intervention, participants will complete a semistructured qualitative interview lasting roughly 30 minutes. The interview probes several domains, including their experience with the program, likes and dislikes, and experience with specific program components (eg, storyline, home practice, materials, and ease of use). Additional questions will relate to participants’ relationship with their Empower Coach.

### Sample Size

A sample size of 70 (n=35 per group) should provide at least 80% power, allowing for a 20% attrition rate for both arms. Specifically, we considered the case where the 2 arms are compared in terms of weekly changes in the mean PHQ-9 survey responses between the baseline, 5 interim in-app PHQ-9 assessments (roughly at weeks 1, 3, 5, 7, and 9), and the 10-week follow-up PHQ-9 survey. We assume that the changes in the mean response can be expressed in terms of a linear trend, and the treatment effect can be expressed in terms of the difference in slopes or rate of change (δ = –0.8 × 5 in the total PHQ-9 score over 10 weeks; 5 is the SD in the change scores in our feasibility study). Sample size estimates used the following inputs: (1) type I error rate (α)=.05; (2) the smallest meaningful difference to be detected (δ) over the 10 weeks; (3) power (γ)=.80; (4) we assumed that outcome within-subject variance (σ^2^=6^2^; estimated from our feasibility study data) is constant over time; (5) the number of repeated measurements per person (n): 1 baseline, 5 in-app assessments, 1 final postbaseline for a total of 7 repeated assessments; and (6) regarding between-subject variation of the slopes, we assumed a variance of random slopes τ^2^=0.2 (estimated from our feasibility study data). The sample size is 52 for both arms with equal randomization probabilities; with an attrition rate of 20% for both arms, the total required sample size is 65. We set the final target sample size to 70 to allow room for higher attrition and lower adherence.

### Analytic Plan

To reduce bias, analyses will involve an intention-to-treat design. Linear mixed models will be used to test within-group changes over time and to compare changes between the treatment and control groups in outcomes of interest. The LASSO (least absolute shrinkage and selection operator [56,57]) variable selection technique will be used to find the optional selection of covariates for the final model. Exploration of mechanisms of change variables involves a procedure described by Hayes [58] that uses bootstrapping to test indirect effects. Statistical analysis will be conducted using Stata 15 SE. Although this trial is not fully powered for mediation analysis, findings from these analyses will inform future research to identify mechanisms of change.

We will use a pragmatic approach involving inductive and deductive coding for qualitative data. Qualitative analysis of the interview data collected from the completed single-group feasibility study is underway, and a codebook will be created during the process. The codebook from the single group study will serve as a framework for us to code the qualitative data collected from the pilot RCT. The codebook will be iteratively refined by reviewing the data and themes several times. Qualitative analysis will be conducted using Dedoose, a web app for mixed methods research.

### Ethics Approval

This study was reviewed and approved by the Health Sciences and Behavioral Sciences institutional review board (IRB) at the University of Michigan. Approval was granted on April 19, 2022 (HUM00212950).

A waiver of documentation for written consent was approved by the IRB. Consent to participate in the study will be obtained orally from participants. Consent will be obtained at 2 points. At first contact, participants will provide consent to be screened. Second, after screening and before the baseline interview, study-eligible participants will be asked to provide oral consent to participate in the study. This will be recorded via a tape recorder after a verbal review of an informed consent document. To ensure the quality of consent, all participants will reconsent at the beginning of the first web-based session of the intervention. This will be accomplished by providing a brief written consent document on the first page of the web-based session. Participants must confirm their understanding of the document to proceed to the first web-based session.

The privacy of participants will be protected in several ways. All participants will complete web-based therapy sessions by themselves in their private homes. Direct identifiers are not stored in the web-based program. Mental health assessment data collected as part of the web-based sessions are stored in a HIPAA-compliant database. Other study data (eg, baseline and posttest assessments) are entered and managed on REDCap. Only the study team members directly authorized by the principal investigator will have access to study data with identifiers. Access is typically restricted to the principal investigator and project coordinators. As the study involves follow-up, direct identifiers will be retained for this purpose. After completion of the study and data cleaning, all direct identifiers will be removed from the database, rendering the final data set anonymous.

Those who qualify for the trial will be paid up to US $100 for participation. Compensation is determined based on the number of assessments completed. Partial compensation is available (eg, participants who complete only the baseline assessment will receive US $30). Participants will be provided US $30 for the baseline, US $50 for the posttest, and US $20 for the follow-up. Payment will be provided directly to the participant via mailed check or Visa gift card, whichever is preferred by the participant.

## Results

Funding for this multiphase project was awarded in November 2020. IRB approval for the single-group feasibility study and the proposed RCT was attained in April 2022. Enrollment for the single-group study began in May 2022, and all subjects completed the intervention by December 2022. Recruitment for the RCT began in January 2023, and the study completion is anticipated by December 2023. The trial is registered at ClinicalTrials.gov (NCT05593276).

## Discussion

### Principal Findings

To our knowledge, this is the first RCT of layperson-facilitated iCBT for low-income homebound older adults with depression. We hypothesize that the Empower@Home intervention will be feasible and acceptable, reduce depressive symptoms, and improve psychosocial functioning and health-related quality of life. Existing evidence-based mental health treatments are often inaccessible to homebound older adults, who have been largely excluded from digital mental health interventions. This trial addresses that gap by testing an intervention specifically designed to the needs of this population.

iCBT participants are exposed to the same components as conventional CBT, though they receive psychoeducational materials through a self-directed internet platform. A meta-analysis of iCBT trials with older adults found a large pooled within-group effect size, although most studies adopted weak designs [24]. In a recent iCBT pilot trial led by the last author, homebound older adults with low computer literacy found that iCBT was an acceptable way of treating depression and resulted in significant reductions in depressive symptoms at the posttest [33]. A recent RCT showed that iCBT also effectively prevented depression among older adults with multiple chronic conditions [29].

As a result of the COVID-19 pandemic, virtual care is gaining rapid traction, which provides unprecedented opportunities for integrating digital mental health interventions into community aging services. The COVID-19 pandemic has accelerated the adoption of technology and virtual care in community settings, which is likely to stay after the pandemic. Although notable age differences in technology use remain, the adoption of key technologies by older adults has grown markedly, and the age-related digital divide continues to narrow. For example, smartphone ownership increased from 46% in 2018 to 61% in 2021 among those 65 years and older [59]. Given the increased focus on digital inclusion, there is likely to be continued growth in access to broadband internet. The Affordable Connectivity Program (formerly Emergency Broadband Benefit), a long-term, US $14 billion program authorized by the 2021 Infrastructure Investment and Jobs Act, provides up to US $30 per month toward internet service and up to US $100 to purchase a computer or tablet device for eligible households. With providers and clients more willing to use technology, increased funding, regulatory changes, and improved technology infrastructure, digital mental health interventions are ripe for implementation in community aging services, where many homebound older adults receive services.

The pilot RCT will provide crucial data for a fully-powered effectiveness trial in the future. If confirmed effective, several probable funding mechanisms can facilitate long-term sustainability. These funding sources include Medicaid, federal grants, private pay, and commercial insurance. For example, community agencies can apply for federal grants authorized by the Older Americans Act that support community-based health-promotion programs. Many commercial insurance providers are already reimbursing for iCBT services [60,61]. Agencies could also charge a sliding scale fee to cover operating costs and raise revenues. In terms of training, most of the provider training materials are web-based, making it relatively easy to scale up.

### Strengths and Limitations

The strengths of this study include an iteratively developed intervention incorporating patient and provider feedback, randomized design, use of laypersons as interventionists, and recruitment of an underserved, hard-to-reach population with high unmet mental health needs. For limitations, this study uses a small sample size and a waitlist control design, which may lead to an overestimation of treatment effects [62]. Though this intervention is intended to address depression in older adults, it may also address related conditions not measured in this study, such as demoralization [63,64].

### Conclusions

The planned study addresses the shortage of iCBT products suitable for homebound older adults. Our treatment innovation, if found effective, will contribute to closing the digital divide and mental health disparities in homebound older adults. Study implications extend to other digital mental health interventions and populations with physical disabilities and access restrictions who face persistent disparities in mental health.

## Abbreviations

CBT: cognitive behavioral therapy
HIPAA: Health Insurance Portability and Accountability Act
iCBT: internet-delivered cognitive behavioral therapy
IRB: institutional review board
PHQ-9: Patient Health Questionnaire-9
PROMIS-GH: Patient-Reported Outcomes Measurement Information System–General Health
PROMIS-SI: Patient-Reported Outcomes Measurement Information System–Social Isolation
RCT: randomized controlled trial

## Data Availability

The data sets generated or analyzed in this study will not be publicly available. Consent and ethical approval for this study does not include a provision for the sharing of data from this study.
